# A novel inhibitor of hypoxia-inducible factor-1α P3155 also modulates PI3K pathway and inhibits growth of prostate cancer cells

**DOI:** 10.1186/1471-2407-11-338

**Published:** 2011-08-05

**Authors:** Sonal M Manohar, Amol A Padgaonkar, Archana Jalota-Badhwar, Vinay Sonawane, Maggie J Rathos, Sanjay Kumar, Kalpana S Joshi

**Affiliations:** 1 Department of Pharmacology, Piramal Life Sciences Limited, 1-Nirlon Complex, Goregaon, Mumbai-400 063, India; 2 Department of Medicinal Chemistry, Piramal Life Sciences Limited, 1-Nirlon Complex, Goregaon, Mumbai-400 063, India; 3 Target Identification Group, Piramal Life Sciences Limited, 1-Nirlon Complex, Goregaon, Mumbai-400 063, India

**Keywords:** P3155, HIF-1α, prostate cancer, PI3K

## Abstract

**Background:**

Hypoxia-inducible factor-1 (HIF-1) is a master regulator of the transcriptional response to hypoxia. It is essential for angiogenesis and is associated with tumor progression and overexpression of HIF-1α has been demonstrated in many common human cancers. Therefore, HIF-1α is one of the most compelling anticancer targets.

**Methods:**

To identify HIF-1α inhibitors, luciferase reporter gene assay under hypoxia and normoxia was used. Detailed studies such as western blotting, RT-PCR, immunofluorescence were carried out to elucidate its mechanism of action. Antiangiogenic activity of P3155 was demonstrated by migration assay and tube formation assay. Efficacy study of P3155 was performed on PC-3 xenograft model.

**Results:**

P3155 showed specific HIF-1α inhibition with IC_50 _of 1.4 μM under hypoxia. It suppressed HIF-1α expression as well as PI3K/Akt pathway and abrogated expression of HIF-1-inducible gene viz. vascular endothelial growth factor (VEGF). P3155 in combination with HIF-1α siRNA showed significant synergistic effect. In addition, it demonstrated significant *in vivo *efficacy and antiangiogenic potential in prostate cancer cell lines.

**Conclusion:**

We have identified a novel HIF-1α inhibitor P3155 that also modulates PI3K/Akt pathway, which may contribute to its significant *in vitro *and *in vivo *antitumor activity.

## Background

Hypoxia is a common phenomenon in rapidly growing solid tumors, and an important microenvironmental factor that contributes to the development of more malignant phenotypes [1,2]. It triggers adaptive responses in solid tumors that include induction of angiogenesis and a switch to anaerobic metabolism [3]. Cells adapt to hypoxia by down-regulating oxygen- and energy-dependent processes, such as mRNA translation or protein synthesis [4] while simultaneously up-regulating specific genes that promote angiogenesis and stress survival.

HIF-1 is a heterodimeric protein and is composed of oxygen sensitive HIF-1α and constitutively expressed HIF-1β/ARNT subunit. Under nonhypoxic conditions, HIF-1α protein is rapidly and continuously degraded by ubiquitination and proteasomal degradation. Degradation of HIF-1α is dependent on binding with von Hippel-Lindau and hydroxylation of Pro-564 via an enzymatic process that requires O_2 _and iron [5,6]. However, under hypoxic conditions, prolyl hydroxylases become inactivated, and thus, HIF-1α is stabilized and HIF-1 activated.

In many human tumors, HIF-1α has been found to be highly expressed due to hypoxia or when oncogenes or tumor suppressor genes are mutated. Immunohistochemical analyses show that HIF-1α is present at higher levels in human tumors than in normal tissues [7]. Furthermore, a correlation between HIF-1α overexpression and resistance to radiation therapy or chemotherapy leading to poor patient prognosis has been observed [8]. Moreover, tumor growth and angiogenesis in xenograft tumors also depends on HIF-1 activity and on the HIF-1α expression [9]. Thus, HIF-1α is viewed as an excellent target for the development of novel cancer therapeutics [10].

Recently we focused our efforts on the development of novel inhibitors of HIF-1α We screened for small molecules to find inhibitors of HIF accumulation under hypoxic condition. In our quest for finding potential therapeutics, we used pyridylpyrimidine as a basic scaffold; various molecules were designed, synthesized and characterized. Out of these molecules, P3155 and P2630 were identified as potent and specific HIF-1α inhibitors in the reporter gene-based assay [ref [11]- structure 7 and 4a respectively]. Herein, we report the anticancer activity and mechanism of action of P3155.

## Methods

### Cell culture

Human prostate cancer cell lines PC-3 and DU145 were cultured in RPMI-1640 containing 10% fetal bovine serum (FBS) (Hyclone, UT, USA), 2 mmol/L L-glutamine (Gibco, Grand Island, NY, USA), 100 U/ml penicillin and 100 mg/ml streptomycin (Gibco). Human umbilical vein endothelial cells (HUVECs) were obtained from Cascade Biologics (Oregon, USA) and were cultured in M-200 medium (Cascade Biologics) supplemented with low serum growth supplements (LSGS) (Cascade Biologics), penicillin-G (100 U/ml), streptomycin (100 μg/ml) and amphotericin B (50 ng/ml) (Gibco). The cell lines were maintained in a humidified incubator at 37°C and 5% CO_2_. Topotecan was purchased from Calbiochem. P3155 was synthesized at Piramal Life Sciences Ltd., Mumbai, India. Both the compounds were dissolved in dimethyl sulfoxide (DMSO) at a concentration of 10 mmol/L (10 mM) and stored at -20°C until use; were diluted in culture medium RPMI-1640 immediately before use and was used within 4 h. All reagents were purchased from Sigma Chemical (St Louis, MO, USA) unless otherwise mentioned.

### Luciferase Reporter assay

U251-HRE and U251-pGL3 cell lines were procured from Dr. Giovanni's Lab and maintained as described by Rapisarda et al previously [12]. Luciferase reporter gene assay was carried out using both these cell lines as described previously in detail [12]. Data was analyzed to determine the EC_50 _concentration (concentration of compound that inhibited luciferase expression by 50%).

### Western blot analysis

PC-3 cells were used for western blot analysis as described earlier [13]. Compounds were added according to concentrations and desferoxamine (DFX) (hypoxia mimick) at a final concentration of 60 μmol/L was added to each of the plates except the control (no DFX) plate. The plates were then incubated in a humidified incubator (5% CO_2_) for 8 h and then harvested. The antibodies used were anti-HIF-1α monoclonal antibody (BD Biosciences, CA), anti-p-Akt473, anti-p-4E-BP1 antibody (Cell Signaling Technology) or anti-β-actin antibody (Sigma).

### Reverse transcription-PCR

For RT-PCR analysis, total cellular RNA was isolated with TRI reagent (Sigma, USA). cDNA synthesis was carried out and PCR was performed on cDNA with 2× PCR master mix (Fermentas, USA) and the corresponding primers. The following primers were used-HIF-1α Forward primer TATGACCTGCTTGGTGCTGA Reverse primer GGGAGAAAATCAAGTCGTGC annealing temperature of 60°C and cycle no. 32. Tubulin: Forward primer TCTGTTCGCTCAGGTCCTTTTGGCC Reverse primer CGTACCACATCCAGGACAGA annealing temperature of 55°C and cycle no. 30. VEGF Forward primer AACTTTCTGCTGTCTTGG Reverse primer TTTGGTCTGCATTCACAT annealing temperature 55°C cycle no. 35. An aliquot of each reaction mixture was analyzed by electrophoresis on a 1.5% agarose gel and the gel images were obtained using Quantity one software (Bio Rad, USA).

### Immunofluorescence

Cells (0.3 × 10^6^) were seeded onto poly-D-lysine coated glass coverslips and incubated overnight. Cells were untreated or exposed to 60 μmol/L DFX for 8 h, in the absence or presence of different concentrations of P3155 and processed for immunofluorescence staining as described earlier [14]. The antibodies used were: mouse monoclonal anti-HIF-1α antibody (BD Transduction Labs, CA, USA), rabbit polyclonal anti-VEGF antibody (Santa Cruz Biotechnology, CA, USA), FITC-conjugated anti-mouse antibody, Cy3-conjugated anti-rabbit antibody (Chemicon International, USA). Images were captured using fluorescent microscope.

### SiRNA mediated RNA interference

Cells were plated in 6 well plates in FBS-free and antibiotic-free media. The cells were transiently transfected with siRNA (HIF-1α-specific siRNA or non-specific siRNA, QIAGEN, USA) using Lipofectamine2000 Transfection Reagent (Invitrogen, Carlsbad, CA) and allowed to stabilize for 24 h, before being used in experiments.

### Migration assay

Cells were seeded at a density of (0.5 - 2.0) × 10^6 ^per well in a sterile 6 well plate. The plates were incubated overnight in humidified CO_2 _incubator (5% CO_2_) at 37°C under ambient oxygen levels for the cells to form a confluent uniform monolayer on the complete surface of the well. The cell monolayer was scraped to create a "scratch" with a pipette tip and the first image of the scratches was acquired. Compounds were added at various concentrations and the plates were further incubated for 24 h. After the incubation, the plate was placed under a phase contrast microscope, reference point was matched, the photographed regions of the first image were aligned and the second image was acquired.

### 3D Gel Endothelial Tube formation assay

The BD Matrigel Matrix (BD Biosciences, USA) was thawed at 4°C overnight on ice. HUVEC endothelial cells were cultured to 60-80% confluence. Endothelial cell suspensions were prepared by trypsinizing the monolayers and resuspending the cells in culture medium with 5-10% serum (used as an angiogenesis promoter). 0.5 - 1 × 10^6 ^cells per 180 μl were added (per well of 24 well plate) of thawed BD Matrigel Matrix. The cells were allowed to adhere for 2-3 h. Subsequently, the compounds were added at appropriate concentrations to the respective wells. Appropriate control (medium control) was employed. Following 24 - 48 h of incubation, the cells were observed under the microscope for tube formation and angiogenesis.

### In vivo studies

The human prostate cancer cells (PC-3) were grown and harvested. Cells were resuspended in saline at 10 million cells/0.2 mL volume and placed on ice. Severe combined immunodeficient mice were injected in 0.2 mL volume s.c. on the right flank and observed daily for tumor appearance. When the tumors attained a diameter of 5 mm, they were randomized into two groups. For control (group I), water was administered every day p.o. for 20 days and, 50 mg/kg P3155 (group II), P3155 solution in water was administered every day p.o. also for 20 days.

### Statistical analysis

Statistical comparison was made using GraphPad PRISM^® ^(versions 3.0 and 5.0, GraphPad Software, Inc., USA) software where Student's unpaired *t*-test was employed. Statistical significance was evaluated by calculating *p *values. Differences where *p *< 0.05 were considered statistically significant. (*p < 0.05; **p < 0.01, ***p < 0.001). Densitometric analysis was carried out using ImageJ software.

## Results

**Figure 1:**
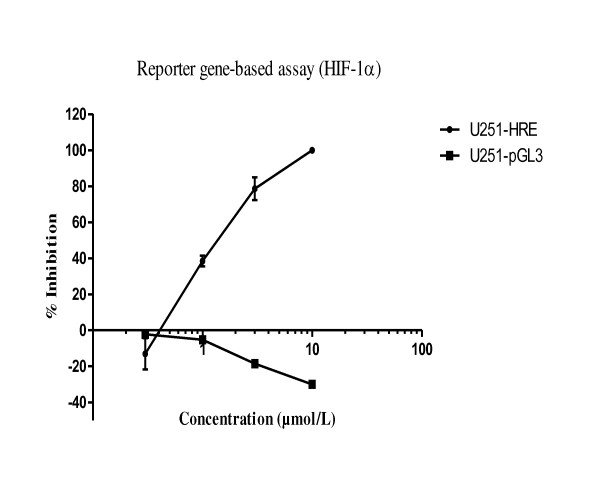
**P3155 specifically inhibits hypoxic induction of luciferase expression in U251-HRE but not in U251-pGL3**. U251-HRE and U251-pGL3 were seeded in 96-well optiplates and incubated under normoxic or hypoxic conditions in the presence or absence of the indicated concentrations (μmol/L) of P3155. Cells were treated for 24 h and then tested for luciferase expression. Results are expressed as percentage inhibition of luciferase levels induced under hypoxic conditions (equal to 100%). Data presented as the average ± SE of three independent experiments.

P3155 was identified by us as a potent HIF-1α inhibitor in the reporter gene based assay [11]. Specificity index (SI) was calculated, obtained by dividing the EC_50 _in U251-pGL3 by the EC_50 _in U251-HRE, which provides an indication of relative specificity towards inhibition of HIF-1 dependent transcription. P3155 had a SI of 7.1, indicating that it had minimal or no activity on the constitutive expression of luciferase in the U251-pGL3 control cell line (Figure 1, Table 1). As HIF-1α protein is overexpressed in PC-3 prostate cancer cell line, it was mainly used for all further studies [15].

**Table 1:** EC_50 _(μmol/L) for P3155 and topotecan (standard) in the HIF-1α reporter gene based assay

**Sr. No**.	Compound No	EC_50 _(μmol/L)	EC_50 _(μmol/L)
		U251-HRE (Hypoxia/DFX)	U251-pGL3 (normoxia)

01	P3155	1.4	> 10

02	Topotecan	0.06	> 3.0

### Effect of P3155 on HIF-1α protein accumulation under hypoxia

**Figure 2:**
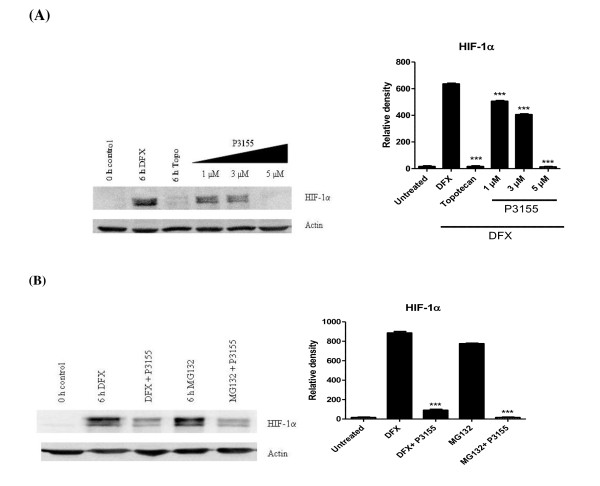
**P3155 specifically inhibits HIF-1α protein accumulation under hypoxia**. PC-3 cells were treated with hypoxic mimic DFX (60 μmol/L) in the presence and absence of P3155 for 8 h, and then protein extracts were prepared as described in 'Materials and Methods' (A) Downregulation of HIF-1α protein by P3155 under hypoxia in a dose-dependent manner (B) P3155 inhibited hypoxia-mediated HIF-1α accumulation in presence of DFX as well as MG132 (proteasome inhibitor) indicating that it inhibits HIF-1α independent of the proteasome activity.

Studies were performed to ascertain the inhibition of hypoxia-induced overexpression of HIF-1α protein in the treated cell samples using western blot analysis. P3155 showed complete abrogation of HIF-1α in PC-3 cell line and was found to inhibit hypoxia-mediated HIF-1α protein levels in PC-3 in a dose-dependent manner (Figure 2A).

### HIF-1α inhibition by P3155 under hypoxia was proteasome independent

HIF-1α is degraded mainly through the proteasomal pathway. To determine whether P3155 treatment induced degradation of HIF-1α via this pathway, we tested the effect of P3155 in presence of 26S proteasome inhibitor MG132 on levels of HIF-1α expression. The level of expression of HIF-1α in the presence of MG132/DFX was upregulated in PC-3 cells as compared with untreated control cells. Simultaneous addition of P3155 with MG132/DFX decreased accumulation of HIF-1α (Figure 2B).

### P3155 downregulates HIF-1α mediated VEGF expression but does not inhibit HIF-1α mRNA levels

**Figure 3:**
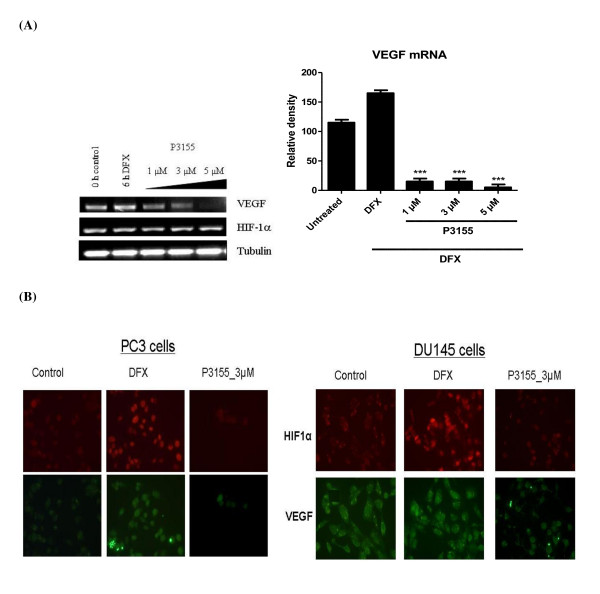
**(A) P276 specifically inhibits VEGF mRNA expression under hypoxia in a dose-dependent manner in PC-3 cells**. However, HIF-1α mRNA levels remained unchanged. (B) Inhibition of HIF-1α and VEGF expression in PC-3 and DU145 prostate cancer cells by P3155. Cells were treated with P3155 (3 μM) for 8 h at 37°C and stained with mouse antibody against HIF-1α and rabbit antibody against VEGF. Reduced coexpression of HIF-1α and VEGF was observed.

Increased HIF-1α expression promotes transcriptional activation of many genes VEGF being a prime candidate. Thus, we determined the levels of VEGF expression that were upregulated by hypoxia-mediated HIF-1 transcriptional activation. P3155 treatment significantly inhibited DFX-stimulated levels of VEGF mRNA. As P3155 was found to act on HIF-1α expression via proteasome-independent pathway, the possibility of it inhibiting HIF-1α transcription was considered. Thus, HIF-1α mRNA levels after P3155 treatment of cells under hypoxia were checked using RT-PCR. P3155 seemed to have no effect on HIF-1α mRNA levels (Figure 3A).

### Effect of P3155 on hypoxia-mediated accumulation of HIF-1α and VEGF

The ability of P3155 to inhibit HIF-1α activity was tested by evaluating levels of transcriptionally active HIF-1α protein expressed in the nuclei of prostate cancer cells PC-3 and DU145 exposed to hypoxia. Treatment of cells with DFX induced accumulation of active HIF-1α protein (Figure 3B red signal) as well as its downstream target VEGF protein (green signal) within the nucleus. P3155 treatment induced a decrease in the co-accumulation of active HIF-1α as well as VEGF protein under hypoxia.

### P3155 inhibits HIF-1α and PI3K signaling pathway under hypoxia

**Figure 4:**
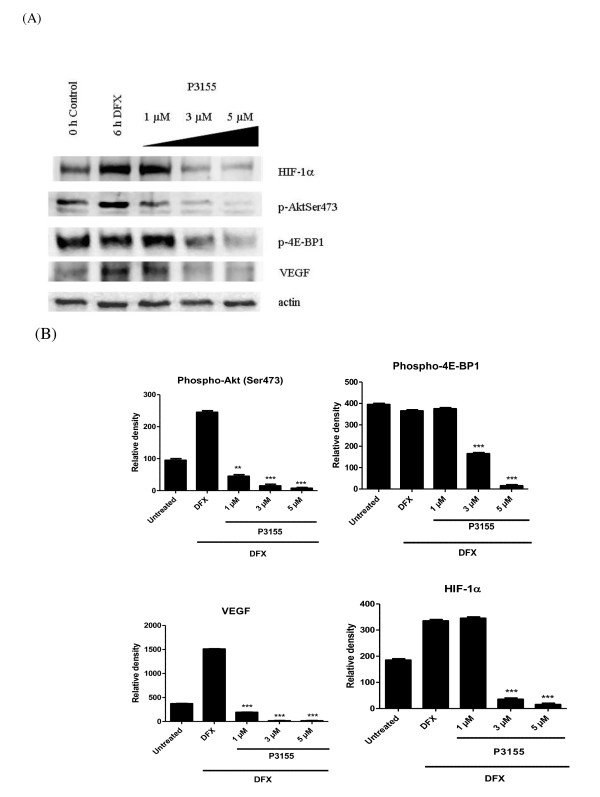
**P3155 downregulates HIF-1α expression as well as PI3K/Akt pathway under hypoxic conditions**. (A) PC-3 cells were treated with given concentrations of P3155 under hypoxia and its effect on protein levels of HIF-1α, phospho-Akt, phospho-4E-BP1 and VEGF was determined using western blot analysis (B) densitometric analysis was carried out for each protein.

Translation of HIF-1α mRNA has been previously suggested to be under the control of the phosphatidylinositol 3-kinase (PI3K) signaling pathway in various cell types [16-18]. To test whether P3155 inhibits this pathway thus probably leading to HIF-1α translational downregulation, effect of P3155 on PI3K pathway was studied using PC-3 in which this pathway is constitutively active due to PTEN mutation [19,20]. PI3K inhibition by P3155 was seen in PC-3 cells as shown by decreased phosphorylation of the downstream targets, Akt and 4E-BP1 besides HIF-1α under hypoxia (Figure 4).

### Effect of P3155 on cancer cell migration

**Figure 5:**
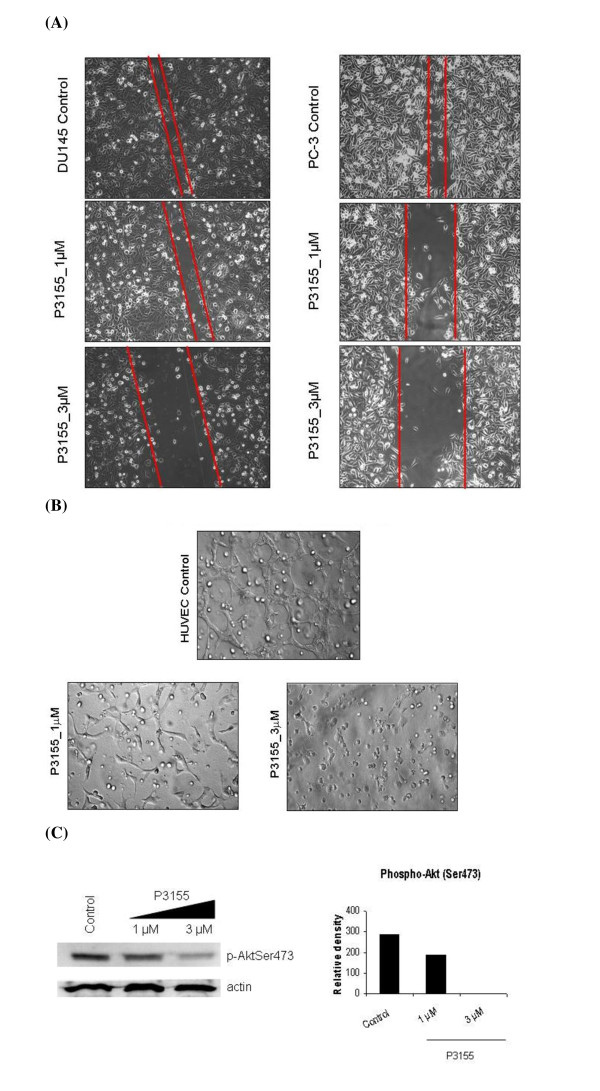
**(A) Effect of P3155 on PC-3 and DU-145 cell migration (B) Effect of P3155 on HUVEC tube formation (C) P3155 downregulates PI3K/Akt pathway as evident by inhibition of phospho-Akt (Ser 473) levels in HUVECs at the same concentrations and timepoint**.

The hallmark of tumor cells is their ability to migrate and metastasize. Prostate cancers are known to metastasize in a high percentage of the cases, which is obviously linked to a poor prognosis. Therefore, potential effect of P3155 on cancer cell migration was tested using the prostate cancer cell lines, DU145 and PC-3. The results demonstrated that P3155 inhibited cell migration of DU145 and PC-3 cells more than two-fold in a dose-dependent manner in the presence of serum (Figure 5A).

### Effect of P276 on tube formation of HUVECs

We next evaluated the effect of P3155 on the formation of functional tubes by HUVECs plated on the Matrigel, a reconstituted extracellular matrix preparation. Serum was used as the stimulator in this study [21]. In the control group stimulated with serum, HUVECs rapidly aligned with one another and formed tube-like structures resembling a capillary plexus within 24 h, which required cell-matrix interaction, intercellular interaction and cell motility. P3155 prevented serum-stimulated tube formation of HUVECs in a dose-dependent manner and no cytotoxicity was observed at these particular concentrations (Figure 5B). At the same timepoint and concentrations, P3155 downregulated phospho-Akt (Ser 473) levels in a dose-dependent and significant manner (Figure 5B).

### Effect of P3155 on tumor growth in vivo

**Figure 6:**
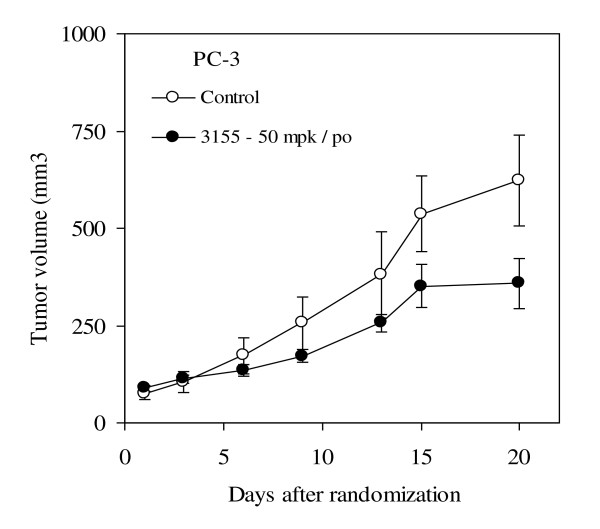
**Effect of P3155 in PC-3 prostate cancer xenograft model**. Mice were treated with daily p.o. doses of P3155 at 50 mpk for 20 days. Percentage tumor weight profile during the course of treatment.

Because of the observed *in vitro *effects of P3155, we investigated whether it inhibits tumor growth *in vivo*. To confirm the *in vivo *activity of P3155, antitumor efficacy of P3155 in xenograft model of PC-3 was studied. The average tumor weights on day 20 for control and P3155 were 933 ± 285.6 mg and 358.6 ± 143.9 mg respectively and the calculated highest percentage of growth inhibition was observed to be 61% on day 9 after randomization (Figure 6, *p *< 0.05). Mice treated with P3155 had no significant weight loss while on the study drug (data not shown). These results indicate that P3155 effectively inhibits tumor growth in tumor bearing mice.

## Discussion

Overexpression of HIF-1α is a common feature of solid malignancies and is correlated with advanced disease stage, increased angiogenesis and poor prognosis [21]. HIF-1 induces expression of genes whose products are involved in cancer cells' survival, glycolysis, angiogenesis, migration and invasion [22]. As a result, HIF-1 has emerged as an attractive molecular target for the development of novel cancer therapeutics. P3155 was identified as a potent and specific inhibitor of HIF-1 transcriptional activation in the reporter gene-based assay. Genetic features of PC-3 prostate cancer cells such as loss of PTEN, constitutive activation of PI3K/Akt pathway, overexpression of HIF-1α, p53 negativity make them an attractive model for studying the mode of action of HIF-1α inhibitors and they have been employed by several groups for such studies earlier [23-25]. Hence, we selected PC-3 cells to elucidate the mechanism of action of P3155 under hypoxia. Since the HIF-1 pathway can be inhibited at several different points, including post-translational modification, gene transcription, or protein translation, next we investigated whether P3155 had any effect on proteasomal degradation of HIF-1α protein, or synthesis of its mRNA or protein. We found that HIF-1α protein downregulation by P3155 is independent of the ubiquitin-proteasomal pathway as blocking P3155-induced HIF-1α protein degradation with the proteasome inhibitor MG132 failed to restore HIF-1α protein expression (Figure 2B). Also our present results indicate that P3155 induced degradation of HIF-1α protein in a dose-dependent manner with no effect on HIF-1α mRNA levels (Figure 3A). Possibility of inhibition of HIF-1α by P3155 at the translational level was therefore considered.

It was previously shown that HIF-1α protein could be stimulated by serum and other growth factors via the phosphotidyl-3-kinase pathway [26]. The PI3K/Akt pathway is activated by hypoxia in certain cell types and interference with the Akt reduces accumulation of HIF in response to hypoxia [27]. This pathway also plays a key role in the control of HIF-1α translation and synthesis in prostate cancer cells. Inhibition of coexpression of HIF-1α and VEGF was observed by immunofluorescence in PC-3 and DU145 prostate cancer cells (Figure 3B). Treatment of hypoxic PC-3 cells with P3155 resulted in reduced HIF-1α expression as well as decreased phosphorylation of Akt and its downstream effector 4E-BP1, which controls the initiation of protein translation under hypoxia [28] in a dose-dependent manner (Figure 4). These observations support the hypothesis that PI3K/Akt pathway could be involved in P3155-mediated HIF-1α inhibition by inhibition of its translation.

Abrogation of HIF-1α and VEGF expression by P3155 was demonstrated by immunofluorescence. In the past few years the field of siRNAs has emerged at a surprisingly high pace. In the present study, we found that P3155 was as effective as HIF-1α siRNA in HIF-1α suppression. Upon transfection with si-RNA, there was dose-dependent inhibition of HIF-1α at the protein level (Additional file 1, Figure S1). It was evident that suboptimal doses of both si-RNA and P3155 resulted in complete abrogation of HIF-1α thus exhibiting synergism.

Tumor invasion and metastasis are essential for tumor growth and are also directly regulated by HIF-1α. Wound healing assays are a classic and commonly used method for studying cell migration and the biology underlying it. The assay has been used for studying cell polarization, matrix remodeling, cell migration as well as a proxy for angiogenesis, metastasis [29]. This assay was employed to assess the anti-migratory potential of P3155. Prostate cancer cells were selected for this study because of their invasiveness *in vitro*. As shown in Figure 5a, P3155 potently inhibited wound healing of these cells and no toxicity was observed. One of the most widely used *in vitro *assays to model the reorganization stage of angiogenesis is the tube formation assay. The assay measures the ability of endothelial cells, plated at subconfluent densities with the appropriate extracellular matrix support, to form capillary-like structures [30,31]. P3155 effectively blocked tube formation by HUVEC cells as shown in Figure 5b in a dose-dependent manner. It also inhibited PI3K/Akt pathway which was evident by decreased phospho-AktSer473 levels. Taken together, these results suggest that P3155 inhibits cell migration and angiogenesis via inhibition of HIF-1α and PI3K/Akt pathway.

A number of anti-cancer agents have been reported to decrease HIF-1α activity in cells in culture however, only a few of the reported HIF-1α inhibitors demonstrated antitumor activity *in vivo *[32,8]. Because of promising *in vitro *results of our identified HIF-1α inhibitor, we examined tumor growth inhibition due to P3155 treatment. The human xenograft model showed efficacy of P3155 in prostate cancer when given by p.o. route. It showed significant anti-tumor activity with 61% growth inhibition. These results suggest that P3155 is an inhibitor of HIF-1 that halts tumor growth by blocking tumor adaptation to hypoxia and thus can be used as a therapeutic modality for aggressive prostate cancer.

## Conclusion

In summary, we conclude from these studies that P3155 is a specific inhibitor of HIF-1α and it also abrogates PI3K pathway. We confirmed the inhibitory effects of P3155 on expression of HIF-1α and on the induction of VEGF under hypoxia by western blot analysis and immunofluorescence. It showed significant antiangiogenic potential on prostate cancer cells and *in vivo*, treatment with P3155 halted the growth of xenograft tumor originating from PC-3 cells.

## Conflict of interests

The authors declare that they have no competing interests.

## Authors' contributions

SMM carried out the cell culture, treatment and mechanistic studies, analyzed the data and wrote the manuscript. AP and AJ designed and performed the migration, tube formation and immunofluorescence experiments. VS performed the *in vivo *experiment. MJR read and edited the manuscript. SK synthesized the molecule. KSJ contributed to the design of the experiment, read and gave final approval of the version to be submitted.

All the authors have read and approved the final manuscript.

## Pre-publication history

The pre-publication history for this paper can be accessed here:

http://www.biomedcentral.com/1471-2407/11/338/prepub

## Supplementary Material

Additional file 1 **Synergistic effect of HIF-1α siRNA and P3155 on HIF-1α expression**. To compare the effect of HIF-1α siRNA and P3155 on HIF-1α protein expression, PC-3 cells were transfected with optimal concentrations of siRNA and P3155 i.e. 20 nM HIF-1α specific siRNA or scrambled siRNA or P3155 (3 μM) under hypoxia. The results showed that hypoxia-induced HIF-1α protein expression was completely suppressed on transfection with HIF-1α siRNA alone or P3155 treatment alone (Figure). Transfection with HIF-1α siRNA combined with P3155, both used at suboptimal concentrations, also resulted in complete abrogation of active HIF-1α expression as shown in Figure. Thus, both these agents when used together showed a synergistic effect. Click here for file
