# Supplementary material for: A novel inhibitor of hypoxia-inducible factor-1α P3155 also modulates PI3K pathway and inhibits growth of prostate cancer cells
Source: BMC Cancer. 2011 Aug 5;11:338. doi: 10.1186/1471-2407-11-338 (PMC3224262; doi:10.1186/1471-2407-11-338)
Supplement: Additional file 1 — Synergistic effect of HIF-1α siRNA and P3155 on HIF-1α expression. To compare the effect of HIF-1α siRNA and P3155 on HIF-1α protein expression, PC-3 cells were transfected with optimal concentrations of siRNA and P3155 i.e. 20 nM HIF-1α specific siRNA or scrambled siRNA or P3155 (3 μM) under hypoxia. The results showed that hypoxia-induced HIF-1α protein expression was completely suppressed on transfection with HIF-1α siRNA alone or P3155 treatment alone (Figure). Transfection with HIF-1α siRNA combined with P3155, both used at suboptimal concentrations, also resulted in complete abrogation of active HIF-1α expression as shown in Figure. Thus, both these agents when used together showed a synergistic effect. [file 1471-2407-11-338-S1.DOC]

*Synergistic effect of HIF-1α siRNA and P3155 on HIF-1α expression*

To compare the effect of HIF-1α siRNA and P3155 on HIF-1α protein expression, PC-3 cells were transfected with optimal concentrations of siRNA and P3155 i.e. 20 nM HIF-1α specific siRNA or scrambled siRNA or P3155 (3 µM) under hypoxia. The results showed that hypoxia-induced HIF-1α protein expression was completely suppressed on transfection with HIF-1α siRNA alone or P3155 treatment alone (Figure). Transfection with HIF-1α siRNA combined with P3155, both used at suboptimal concentrations, also resulted in complete abrogation of active HIF-1α expression as shown in Figure 5. Thus, both these agents when used together showed a synergistic effect.

**Figure S1:** Inhibition of HIF-1α expression by P3155 in PC-3 cells after 6 h of treatment. (A) Cells were treated without or with 3 µmol/L P3155 or 20 nM HIF-1α siRNA under hypoxia and then stained for detection of HIF-1α protein by immunofluorescence. (B) Cells were treated with HIF-1α siRNA or P3155 under hypoxia at the indicated concentrations in the figure. Blue: DAPI (nuclear stain) Red: HIF-1α. Images were captured using fluorescent microscope.

(A)


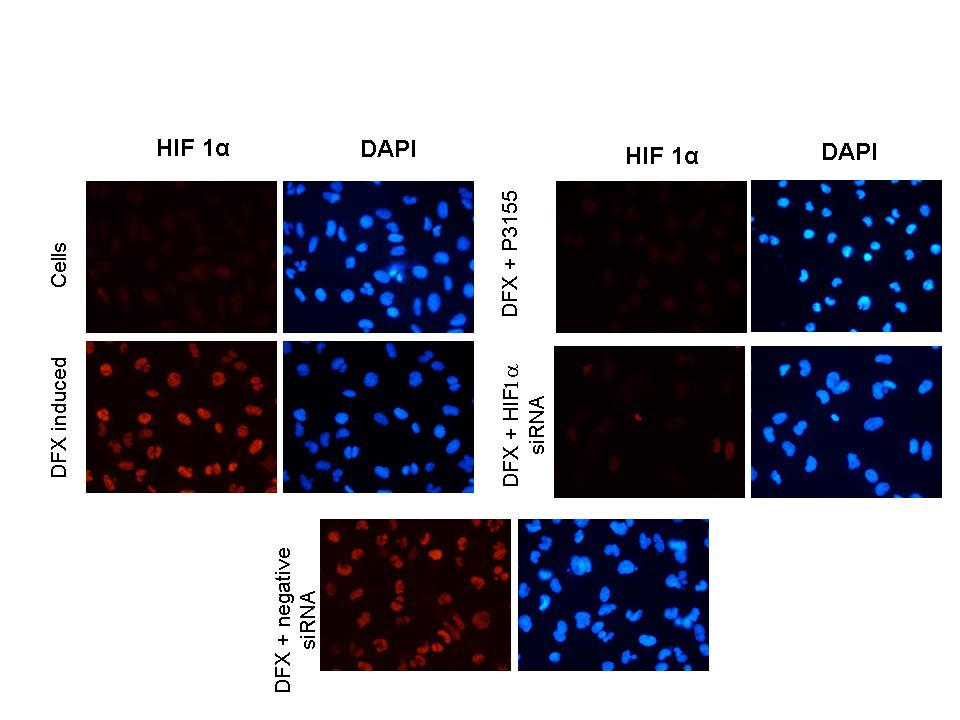


**(B)**

**
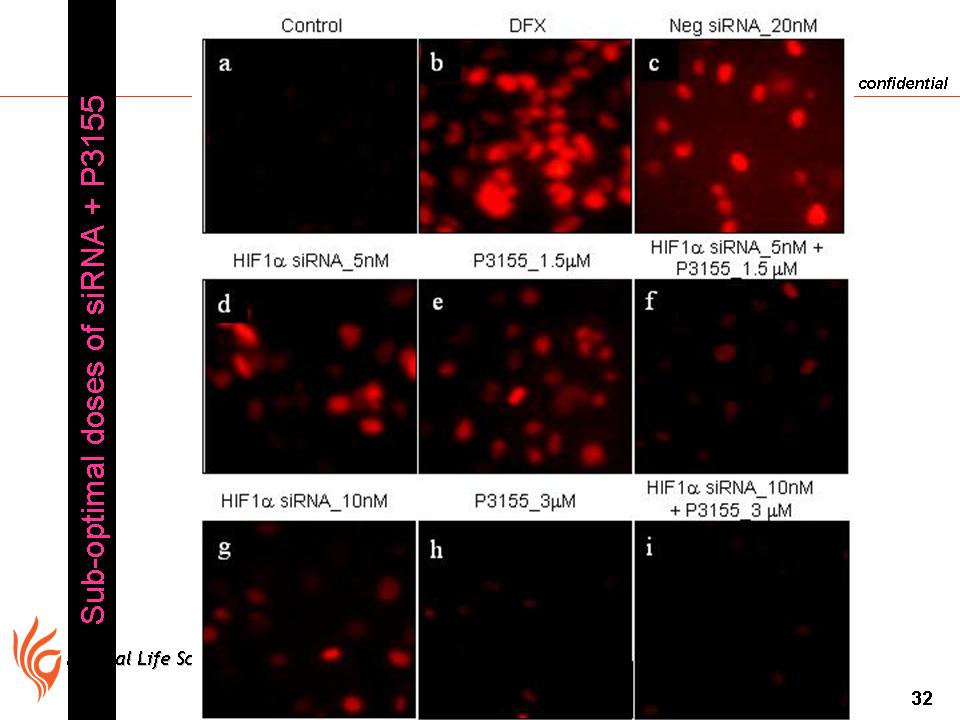
**
